# War, immigration and COVID-19: The experience of Afghan immigrants to Iran Amid the pandemic

**DOI:** 10.3389/fpsyt.2022.908321

**Published:** 2022-07-28

**Authors:** Homa Mohammadsadeghi, Solmaz Bazrafshan, Negar Seify-Moghadam, Golnaz Mazaheri Nejad Fard, Maryam Rasoulian, Mehrdad Eftekhar Ardebili

**Affiliations:** ^1^Psychiatry Department, Medical School, Iran University of Medical Sciences, Tehran, Iran; ^2^Psychology Department, Shahid Beheshti University, Tehran, Iran

**Keywords:** war, immigration, COVID-19, Afghan, Iran

## Abstract

**Introduction:**

Afghanistan's domestic upheaval following the Taliban's invasion leads to massive displacement of its population. The number of Afghan refugees in Iran has dramatically increased since the Taliban's takeover of Afghanistan in August 2021. Multiple pre-and post-migratory traumatic experiences affect immigrants' physical, psychological, social, and economic wellbeing. The coronavirus outbreak, considered a traumatic experience in human life in the 21st century, added to their problems in Iran and exposed them to new challenges. This qualitative study aimed to investigate their experiences early before, during, and after immigration and the pandemic's challenges to their lives in Iran.

**Methods:**

In the present qualitative study, ten Afghan residents living in Iran who immigrated to Iran legally or illegally since the summer of 2021 and the last year after the second Taliban invasion were selected via purposive sampling. A semi-structured interview was applied to gather the data, and the data were analyzed through Braun and Clarke's thematic analysis method.

**Results:**

Ten male participants with a mean age of 26 y/o were interviewed. Their residence in Iran was between 20 days and 8 months. Four main themes were extracted. The first theme, the *Tsunami of suffering*, represents a disruption of the normal flow of life. Six subthemes, including loss, being near death, insecurity, sudden hopelessness, leaving the country involuntarily, and reluctance to explore underlying emotions, are included in this category. The second one, *Lost in space*, describes the participant's attempt to leave Afghanistan following the extensive losses and violent death threats. Their experiences are categorized into four subthemes: the miserable trip, encountering death, life-threatening experiences, and being physically and verbally abused. The third theme, with its five subthemes, try to demonstrate the participants' experiences after getting to their destination in Iran. The last one, *Challenges of the COVID-19* explained the experience of Taliban return, war trauma, running away, and living as a refugee or immigrant coincided with the COVID pandemic.

**Discussion:**

Our interviewees explained multiple and successive traumatic experiences of war, migration, and the pandemic. The central clinical features of survivors are fears of losing control, being overwhelmed, and inability to cope. They felt abandoned because not only lost their family support in their homeland but could not also receive support in Iran due to the pandemic-related social distancing and isolation. They were dissociated and emotionally numb when describing their experience, which is a hallmark of experiencing severe, unprocessed traumas.

**Conclusion:**

Gaining a better understanding of Afghan refugees lived experiences may help provide them with better social and health care support. Proper mental and physical healthcare support and de-stigmatization programs may reduce the impact of multiple traumas on their wellbeing.

## Introduction

Taliban emerged in Afghanistan in the mid-1990s following the withdrawal of Soviet troops, the collapse of Afghanistan's communist regime, and the subsequent breakdown in civil order. This domestic upheaval leads to massive displacement of its population (1). Afghan refugees comprise the largest refugee population in the world (2). The number of Afghan refugees in Iran has dramatically increased since the Taliban's takeover of Afghanistan in August 2021. About five thousand people arrive daily after the takeover, compared to two thousand people beforehand (3). Iran and Pakistan have been the primary host of Afghan refugees for over three decades (1). According to the most recent numbers from October 2020, 2,250,000 undocumented Afghans live in Iran out of 3,636,000. At the same time, 586,000 Afghans have passports, including those on student and extended family visas. Additionally, 780,000 Afghans are refugees (3).

Migration can be a psychological and physical trauma, mainly when it occurs forcefully and involuntarily. People flee to save their lives. It is inevitable, unavoidable, and is not their choice. Multiple pre-migratory stressors such as war, violence, insecurity, torture, murder, homelessness, and starvation force them to leave their home country (4–6). They are looking for a place where they could have opportunities to experience a peaceful and safe present and possible future (7). This unsafe and involuntary migration affects immigrants' physical, psychological, social, and economic wellbeing (8).

It is optimistic if we think it is the end of their miseries. There was frequent physical and psychological trauma along the way, and they experienced many difficulties in the host country (4). After migration, new problems would emerge once they arrive in the new place, and they should struggle with finding work, accommodation, stigmatization, health issues, and multiple losses (2, 6). Because of all these pre-and post-migratory traumatic experiences, they are vulnerable to developing severe mental disorders, post-traumatic stress disorder, mood disorders, and anxiety disorders (2, 9).

The coronavirus outbreak is also a traumatic experience in human life in the 21st century. It seriously impacts mental and physical health, economic and social conditions, and it has revealed that the human being is biologically vulnerable and fragile (10). COVID-19 was first identified in Wuhan, China, in Dec 2019, then spread rapidly to other countries (11). on February 19, 2020, the first cases of Covid-19 were reported in Iran (12). Until March 25, 2022, the disease has infected 7,145,877 people in Iran, and unfortunately, 139,865 of them have died (13).

The disease's unclear and unpredictable nature, the pandemic's unknown end time, and the seriousness of the condition were the major concerns that produced anxiety and disappointment. Social and interpersonal communication has been restricted, and family conflicts have increased due to home quarantines (12).

Refugees are a highly vulnerable subgroup of the population and are at higher risk in the pandemic (14). COVID-19 added to Afghans problems in Iran and exposed them to new challenges (15), Such as poor socioeconomic conditions and accessibility to health care services (14). While the importance of family support to psychological wellbeing is undeniable, Afghan immigrants have also lost this support system through forced relocation, disrupting tight family bonds and socialization during the pandemic (2).

There is not much research available to address this specific issue of an Afghan refugee living in Iran during the Taliban invasion and the pandemic. In this qualitative study, we explored their lived experience early before, during, and after the immigration and the pandemic's challenges to their lives.

## Methods

In this qualitative research, the study population included Afghan residents living legally or illegally in Iran who have immigrated to Iran legally or illegally since the summer of 2021 and during the last year after the second Taliban invasion. We selected the participants *via* the purposive sampling method, which continued until data saturation. The inclusion criteria consisted of age (at least 18 years old), fluency in the Persian language, having at least a high school diploma, no current drug abuse (not in the period of withdrawal or intoxication), and voluntarily acceptance to participate in the study. The exclusion criteria were a history of serious medical diseases such as cancer, diabetes and heart disease, the history of psychiatry and neurological disorders that interfered with the interview. At the beginning of the interview, we asked about the exclusion criteria.

The primary data collection tool was the semi-structured interview with participants. First, the interview guide was prepared, and then the text of the questions was read by several experienced experts. Questions were designed to be open-ended with a focus on the topic in the form of an interview guide. The interview guide ensured that the same information was obtained from all participants.

Initially, Afghan immigrants who wished to share their experiences with the researchers were selected among the available individuals regarding the inclusion and exclusion criteria. Before interviewing, the aim of the study was explained to the volunteers, and the confidentiality issues were discussed. The permission to record the interview was obtained concerning the proper protection of the audio documents. The participants ensured that the information would be applied just for the research without revealing their identity. The right to leave the interview at any time was among the other ethical considerations of the present study. The individuals then entered the process of a 1-h interview. During the interviews, there were two facilitators, one conducting the interview and the other recording the participants' feelings and reactions. Data collection continued to acquire relative saturation. Eventually, ten individuals were interviewed.

For managing, organizing and analyzing the data, Braun and Clarkes thematic analysis method (16, 17) was used. The recorded interviews were first transcribed in Word software for the content analysis. Then, the text was read several times, and the meaning units were extracted to understand the interviews' content in line with the research question. The codes were summarized and classified according to their similarity in the following. The information obtained was then discussed in meetings with the research team.

In order to evaluate the validity, credibility, and dependability of the research findings, two review methods, member check and peer reviewers check, were applied. After completing the data analysis, the findings were checked with the individuals who participated in the study. The data were analyzed again by another expert and compared to the analysis results of the researchers of the present study.

### Ethics

The study was approved by the Mental Health Research center at the Iran University of Medical Sciences (IUMS). All participants filed an informed consent form reviewed and approved by the Ethics Committee of IUMS. The researchers keep the names of the participants confidential and do not disclose information that may lead them to be recognized.

## Results

Ten male participants aged 19 to 45 (mean age = 26) were interviewed. Their residence in Iran was between 20 days and 8 months. Six participants were refugees, and 4 were immigrants. Four were single, and the rest (6) were married.

### Tsunami of suffering

This theme represents widespread changes, disruption of normal flow of life, and impending danger of being murdered, which leads to widespread community fear. The changes took place rapidly and affected their life negatively in different ways; many aspects of their lives were influenced. The experiences are represented in six subthemes: loss, near death, insecurity, sudden hopelessness, leaving the country involuntarily, and reluctance to explore underlying emotions. The last subtheme is derived from interviewer notes and was seen in all participants; and reaffirmed by supplementary interviews with another interviewer. Table 1 shows the sub-themes, codes, and quotations of the Tsunami of suffering theme.

**Table 1:** Sub-themes, codes, and quotations of the Tsunami of suffering theme.

**Sub-themes**	**Codes**	**Quotations**
Loss	Becoming jobless	I was engaged in agriculture; I had my own shop. We would live on our own land. We abandoned all. All ran away.
		I was a welder working for the Afghanistan military in the repair and maintenance section. When the Taliban rose to power, they closed that section.
	Becoming unhealthy and injured	They bombed our station. My back was injured. Now I have limitations in work.
	Becoming homeless	Taliban Attacked our home several times. Our home was destroyed.
Being near death	Witnessing murder	We encountered the Taliban, and a bullet hit me. My brother was murdered.
	Violence	The Taliban would not let soldiers of the previous government stay alive. Governmental agents and people collaborating with the previous government were hit, imprisoned, and killed.
	Home under fire	Taliban would come near our house, shooting at it. We were at home while bullets hit our house. Our house was ruined.
	Torture	They hit my father several times to tell them my place.
Insecurity	Live secretly to save life	I would live secretly and would change my place frequently. There was a fight for life. Many Afghans saved money in banks. When the war began, banks closed instantaneously. Anyone who had money in banks lost it.
	Life threats	I was given a murder sentence both by phone and face-to-face in the attack on our home. They told me I was Shia and I should be the follower of true Islam again and start over.
	Economic insecurity	Many Afghans saved money in banks. When the war began, banks closed instantaneously. Anyone who had money in banks lost it.
Sudden hopelessness	Hopelessness	A kind of mental disorder afflicted all. All people's hopes gave way to hopelessness. When hope is gone, nothing remains except anarchy; people are just alive by hope. A huge wave of hopelessness suddenly broke on the people, especially young ones; they were suddenly hopeless about the future.
Leaving the country involuntarily	Abandoning the country to save the life	If I had not had to come to Iran, I wouldn't have been here. Who abandons his own home? Only God knows what I feel. I came here just to save my life.
	Economic reason for leaving	I was a tailor. After beginning the war, the people no longer had money to make clothes. I had to come to Iran.
Reluctancy to explore underlying emotions	Not willing to talk about feelings	Interviewer's note: He changed the topic after I asked him to talk about his emotional reaction to war trauma. Interviewer's note: I tried encouraging him to talk about his feelings, but I failed.

### Lost in space/dangerous escape to Iran

The participants attempted to leave Afghanistan after extensive losses and violent death threats. However, this was not easy. They had to leave the country secretly and illegally and with the help of human traffickers. They had to tolerate misery in Afghanistan and Iran and take considerable risks on this journey. The participants' experiences in this trip are categorized into four subthemes: the miserable trip, encountering death, life-threatening experiences, and being physically and verbally abused. Table 2 shows the sub-themes, codes, and quotations of the Traumatic Escape theme.

**Table 2:** Sub-themes, codes and quotations of the Traumatic Escape theme.

**Sub-themes**	**Codes**	**Quotations**
The miserable trip	Crying for misery	I suffered real misery on my journey to Iran. It was a fate worse than death. I had been crying every day, complaining to God about my condition.
	Hunger	We had to walk for hours on the border of Iran. We were suffering from hunger.
Encountering death	Feeling of being struck by a bullet	After crossing the border, an agent shot me just before getting to Iran-Shahr; we ran away; I supposed I was struck. Then I called the trafficker; he became angry about why we went far away and insulted me by saying obscene words.
	Being shot	… we went to the (Tajikistan) border; we slept on the plain, we struggled to cross the border, but we couldn't. Tajikistan's agents were shooting us. Many were hit and killed there.
Life-threatening experiences	Ready to die	You should be ready to die. We sit in a car, and if you say anything, you are done.
	Risk of death	16–18 people got into a sedan car to pass cities and the border. The car could be fired a volley of bullets. Drivers smoke heroin or opium. They are high and drive recklessly over 160 km/hr.
Being physically and verbally abused	Being beaten	We passed Pakistan's border hardly. We were 35 people who got on a Toyota pickup. The trafficker would yell continuously at us to sit down. I said my feet were broken. We were hit and insulted a lot there. If you say anything, they use dirty words and bit you.
	Harassment	I was interrogated about where I had come and what I had been doing for my life to make sure I was not from Isis. They looked in my bag and pockets. They upset you. They insult you. They empty your bag. There were both Taliban and Isis on the way. They stopped us. Hit us and took our money. If you say you are Shia, they take you and hit you. They hit. They kill.
	Cruel behavior	We were in the trunk of a Peugeot. Once I asked him to pull back the black cloth because I was unable to breathe. We were in a small and closed space. He said lots of obscene words. I felt I was dying.

### From being a citizen to being a refugee

This theme is about the participants' experiences after getting to their destination in Iran. The related data are categorized into five sub-themes: stigma, awful condition of employment and difficulties in getting them, being away from one's home and family, support of compatriots and friends, and reasons for staying in Iran.

The stigma sub-theme describes how the participants are viewed and treated differently because of their nationality. The second sub-theme, related to stigma, represents the participants' struggles to find a job and their particular problems in the workplace. One significant misery of them is being away from their families. Some of them did not have any news from their family. Amongst these stresses, the help and support of a friend or compatriot play a significant role in spending the initial period on finding a job and adapting to the situation. The last sub-theme explains that they endure the condition and stay here despite their complex condition in Iran. Table 3 shows the sub-themes, codes, and quotations of the From Being a Citizen to Being a Refugee theme.

**Table 3:** Sub-themes, codes and quotations of the From Being a Citizen to Being a Refugee theme.

**Sub-themes**	**Codes**	**Quotations**
Stigma	Abuse	If you come to Iran from Afghanistan, you must put your life on the line. They treat you like a sheep, hit you, take your money, get your right, and if you say anything in return, you are fired.
	Looking different	On this trip to Iran, I have another feeling. People look at me differently.
	Rejecting attitude	No one let us enter his home when he knows we are Afghan.
	Verbal and physical punishments in camps	The Police arrested me for not having a visa, and I was taken to … camp. I saw a non-human situation. They would hit all severely and use obscene words.
	Humiliation	You are not called by your name in all cities across Iran, such as Tehran, Isfahan, and Shiraz. They use ‘this’ to call you instead. If you go to other countries, you are respected. Iranians do not even mention your name.
Awful condition of employments and difficulties in getting them	Unstable jobs	There was construction work. I worked for 2 days, and then they told me they did not need me anymore. I sought a job and went somewhere, but I did not find any. They find an excuse to fire me. They criticize me, saying I do not have skills. They find a justification to expel you.
	Abuse by employer	I used to weld in Tehran. My employer postponed my salary several times. My brother had a car accident. I called my employer four times and told him I needed money. I asked him to give me a part of it. But he didn't. In the end, I gave up.
	Limitations	As I don't have a visa, I can't work, go out, do any recreational activities, or take a trip.
Being away from one's home and family	Not in contact with family	I haven't heard anything from my family. The home's telephone is off. I called someone and asked him to take the phone to my family, but the attempt was not successful.
	Away from family	I am far away from my wife and children, and this is the most difficult thing. I feel miserable. I am always sad. Why we Afghans are so dejected. We can't spend a couple of good days with our families.
Support of compatriots and friends	Support of a friend	I stayed at my friend's place. He helps me. I will stay there. He will help me to find a job.
	Financial support	I got money from friends and acquittances before finding a job. It took 10 days to find a job.
	Sheltered by friends	My townspeople sheltered me; they worked for some small companies and had dorms. I stayed with them. Only my friends helped me with the costs of living.
Willingness to stay in Iran	Saving life	I was saved from the Taliban's threats.
	Sending money	I work here and send money to my family in Afghanistan.

### Challenges of the COVID-19

The experience of Taliban return, war trauma, running away, and living as a refugee or immigrant coincided with the COVID pandemic. The first sub-theme shows the participants' beliefs that COVID-19 was not a priority in their country and had to deal with more critical problems. They got used to hearing the death of people without doing something against it. The second sub-theme is about failures of the health system against COVID-19 both in Iran and Afghanistan. The participants' problems in getting vaccinated in Iran are represented as the third sub-theme. Due to the restricted social network of the participants in Iran, the restriction imposed by COVID-19 had a significant effect on them and led to more social isolation. Table 4 shows the sub-themes, codes, and quotations of the Challenges of the COVID-19 theme.

**Table 4:** Sub-themes, codes and quotations of the COVID-19 sub-theme.

**Sub-themes**	**Codes**	**Quotations**
Emotional numbness to COVID-19	Less lethal than other threats	People believe we face a more serious crisis than COVID-19. The emergence and coming to power of Takfiri is more lethal than COVID-19. Moreover, they do not fear anything when they are hungry. Currently, there are grave problems, terrible famine, and poverty in Afghanistan to the extent that nobody fears COVID-19. COVID-19 is a forgotten subject. No one adheres to preventive protocols.
	Many people died	In just 1 month, 70 people died because of COVID-19 in my neighborhood. There was a hospital but didn't have good doctors. There was war, and doctors wouldn't go to hospitals. Life was difficult. Every day we used to hear the news of the death of someone. Three of my cousins died…my wife and my mother got sick. I got COVID-19 too. I wasn't able to walk for 10 days.
Healthcare system failures to face with COVID-19	Weak health system	Coronavirus consequences are more severe in Afghanistan However; doctors are weaker; hospitals are more inefficient, there are problems with medication supply, and the fatality rate is higher.
Access to vaccine in Iran	Getting vaccinated	The situation was different here in Iran. Refugees who have come with passports and visas gain some advantages. They were able to get vaccinated. Those who had entered Iran illegally did not have access to any services and vaccines. They were afraid of being arrested by Police and expelled.
Social distancing caused social isolation	COVID limitation	One of my Afghan friends told me he was eager to invite me to his home, but he couldn't because of Coronavirus.

## Discussion

This study explored the inner experiences of war induced sudden breakdown of the lives of Afghans, the traumatic escape, a change from being a citizen to a refugee, and their reciprocal interaction with the worldwide challenges of COVID-19 pandemic. War is an objective and massive trauma that severely impacts individuals' mental and physical health. While Afghan people fled to save their lives after the Taliban currently took over the country, they had challenging experiences during and after their migration. Iran has been a host country for Afghan immigrants and refugees for the last three decades. However, during the pandemic crisis, the experience of relocation was more complicated.

Interviewing ten Afghan immigrants, ***the Tsunami of suffering*** was the main theme of their lived experience in recent years. They lost their job, home, and physical wellbeing. The survivors who accepted to interview were near death; they witnessed murder, violence, torture, and their home under fire. They felt insecure and hopeless and forced to leave the country either as a refugee or by holding a Visa. We found that they were reluctant to express their emotions, a hallmark of being seriously traumatized.

We found another main theme; the ***feeling of being lost in space*** while they had to escape to Iran. They explained their miserable trip and encountering death in their journey. They experienced life-threatening events and were physically and verbally abused.

The third main theme was the ***feeling of transforming from a citizen in their homeland to a refugee in their host country***. They felt discriminated against and stigmatized. They explained the awful condition of employment and difficulties in maintaining their job, resulting in hopelessness and depression. They also described the vital support of their compatriots and friends. Their main reasons for staying in Iran were saving their lives and sending money to their families in Afghanistan.

The final theme was ***challenges of the COVID-19***, which, surprisingly, felt like nothing to them. They were primarily numb to COVID-19. However, they explained the healthcare system's failures to face COVID-19 and the difficulties accessing vaccines in Iran. According to the quarantines, they could not receive enough support from their families and friends in Iran and felt socially more isolated.

A single trauma can result in mental disorders, but multiple and chronic cumulated traumas disrupt a person's mental integrity and dissociative self-state (18). Our interviewees explained multiple and successive traumatic experiences of war, migration, and the pandemic. They were dissociated and emotionally numb when describing their experience, which is a hallmark of experiencing severe, unprocessed traumas.

The threats to one's survival provoke anxieties that reflect concerns over survival, self-preservation, and safety (18). The central clinical features of survivors are fears of losing control, being overwhelmed, and inability to cope. In addition, the feeling of entrapment and sense of disintegration of self, emptiness, humiliation, and fears of abandonment or need for support may complicate the condition (18). Our interviewees experienced a tsunami of suffering, including fears, entrapment, and disintegration of their selves. They felt abandoned and not only lost their family support in their homeland but could not also receive support in Iran due to the pandemic-related social distancing and isolation.

The findings of our study were consistent with another study that determined a high prevalence of discrimination, including health disparities for immigrants in the host countries (19). Our interviewees described their difficulties, including discrimination in access to vaccines in Iran amid the pandemic.

Based on another study (20), immigrants feel more sadness, depression, and loneliness several years after immigration than when they initially arrived. Challenges like lower income, acculturation and ethnic identity, and discrimination make them more vulnerable over time (18). Afghan refugees in Iran are a significant minority group vulnerable to physical and mental disorders. Enhancing their wellbeing in Iran during the following years requires precise and delicate planning. Providing proper mental and physical healthcare support and de-stigmatization programs are suggested.

One of our methodological limitations was related to the nature of the traumatic experience itself. Our interviewees were dissociated and emotionally numb when describing their experiences; we know this is a hallmark of experiencing severe, unprocessed traumas. However, these psychical defense mechanisms may be a barrier to a depth interview in the qualitative study. In addition, it was difficult for some interviewees to trust the interviewer. Because of their illegal residence in Iran, they refuse to give detailed information about themselves.

Since we had not had any other study in this area in Iran before, the finding of this study should be regarded as preliminary and suggestive. We need more study on more comprehensive ranges of people and include other resources such as writing and arts. Moreover, a data triangulation approach is needed in future studies.

According to the finding of this study, it is crucial to deliver primary care and mental health services to Afghan immigrants regardless of their immigration documents. Health systems can give services with their national ID and ensure that their data will not be used to recognize or expel them. Simultaneously, health professionals should be trained to be familiar with their specific problems.

## Conclusion

We sought to develop a richer understanding of the experiences of Afghan residents in Iran during the Taliban's dominance in their country amid the pandemic. Multiple pre-and post-migratory traumatic experiences affect immigrants' physical, psychological, social, and economic wellbeing. The coronavirus outbreak also complicated their situation. They encounter disruption of the normal flow of life due to loss, being near death, and insecurity. Their miserable trip, life-threatening experiences on their way to Iran, their difficult life situation in Iran, and the challenges of the COVID-19 had worsened the situation. Providing social and health care support in Iran may reduce the impact of multiple traumas on their wellbeing.

## Data Availability Statement

The raw data supporting the conclusions of this article will be made available by the authors, without undue reservation.

## Ethics Statement

The studies involving human participants were reviewed and approved by Mental Health Research Center. The patients/participants provided their written informed consent to participate in this study.

## Author contributions

HM, MR, NS-M, and GM made substantial contributions to the conception and design of the work. SB had a substantial contribution to data gathering. ME analyzed and interpreted the data. All authors were contributors to writing the manuscript and read and approved the final manuscript. All authors contributed to the article and approved the submitted version.

## Conflict of interest

The authors declare that the research was conducted in the absence of any commercial or financial relationships that could be construed as a potential conflict of interest.

## Publisher's note

All claims expressed in this article are solely those of the authors and do not necessarily represent those of their affiliated organizations, or those of the publisher, the editors and the reviewers. Any product that may be evaluated in this article, or claim that may be made by its manufacturer, is not guaranteed or endorsed by the publisher.
